# The Benefits of Water from Nitrodi’s Spring: The In Vitro Studies Leading the Potential Clinical Applications

**DOI:** 10.3390/ijms241813685

**Published:** 2023-09-05

**Authors:** Ilaria Mormile, Fabiana Tuccillo, Francesca Della Casa, Valentina D’Aiuto, Nunzia Montuori, Marina De Rosa, Filomena Napolitano, Amato de Paulis, Francesca Wanda Rossi

**Affiliations:** 1 Department of Translational Medical Sciences, University of Naples Federico II, 80131 Naples, Italy; ilariamormile87@gmail.com (I.M.); francescadellacasa4@gmail.com (F.D.C.); nmontuor@unina.it (N.M.); depaulis@unina.it (A.d.P.); francescawrossi@gmail.com (F.W.R.); 2 Department of Law, University of Naples Federico II, 80138 Naples, Italy; fabiana.tuccillo@unina.it; 3 Post-Graduate Program in Clinical Immunology and Allergy, University of Naples Federico II, 80131 Naples, Italy; valedaiuto@tiscali.it; 4 Center for Basic and Clinical Immunology Research (CISI), WAO Center of Excellence, University of Naples Federico II, 80131 Naples, Italy; 5 Department of Molecular Medicine and Medical Biotechnology, University of Naples Federico II, 80131 Naples, Italy; marina.derosa@unina.it

**Keywords:** Nitrodi, spring, thermalism, wound healing, balneotherapy, water, Romans, spa

## Abstract

Natural products (water, plants, and minerals) have been studied for diverse applications in health and disease. Since there has been a growing interest in the introduction of thermal water as a clinical complementary approach in the treatment of low-grade inflammation and stress-related conditions, this review focuses on the oldest spa in the world: Nitrodi’s spring. Substantial studies in the 1960s showed that both the internal and external use of Nitrodi’s water yielded several benefits in physiological processes and in treating certain disorders, mainly allergic and autoimmune inflammatory conditions. More recently, a novel interest in Nitrodi’s water has prompted researchers to further explore the effects of this water and shed light on the molecular mechanisms sustaining its therapeutic efficacy. In different epithelial cell models, Nitrodi’s water had strong promotional effects on proliferation, cell migration, cell viability, and fibroblast to myofibroblast transition, all of which essential for wound healing and tissue remodeling. Moreover, Nitrodi’s water exhibited anti-oxidant and anti-inflammatory properties through the inhibition of ROS production and protein S-nitrosylation. Here, we have collected the clinical and basic data on Nitrodi’s water and reviewed articles that have discussed its use as a potential treatment for several inflammatory and autoimmune diseases and age-related skin deterioration.

## 1. Introduction

### 1.1. Baths in the Roman Culture

In the Mediterranean Region, the exploitation of thermo-mineral water springs is a traditional phenomenon [1]. Since ancient times, water has been associated with positive effects on health, strengthening its relationship to humankind [2]. Herodotus spoke of the divinity of water and its potential ability to heal all diseases, including blindness, deafness, and dumbness [3]. In Italy, an ample number of springs that supported bathing establishments and infrastructures those date back to the pre-Roman age left deep and, in some cases, still well visible marks that often represent the basis of modern thermo-mineral resorts.

Campania is one of the richest regions with respect to thermal and mineral water resources [4]. It may be said that Romans revalued thermalism. In the Roman age, bathing was a social and recreational activity deeply rooted in daily life. There are numerous waters with special chemical and physical properties and declared healthful features and, since Roman times (VIII–V centuries BC), these waters have been used by humans for therapeutic purposes. Thermalism was developed by the Romans by first building waterworks and thermal structures in the cities and then large spas in Baia, Campi Flegrei, Agnano, Ischia [5], and many other locations that have been highly praised for curing a wide range of pathologies due to the volcanic origin of their waters and their diversified physico–chemical and thermic properties [6,7,8,9,10,11,12,13,14,15,16,17,18,19].

In Nitrodi’s spring, already known to the first Greek settlers in southern Italy and dedicated to Apollo and the Nitrodi Nymphs, everyone found a remedy—war wounds healed in a short time, old men became looser, and women emerged from the water more beautiful. Thus, the thermal waters of Nitrodi became a very important cult center between the 1st century BC and the 3rd century AD. The healing power of Nitrodi’s water was considered a gift from the nymphs and the god Apollo. As evidence of that ancient cult, today we can admire the votive reliefs in the Archaeological Museum of Naples, found in Nitrodi in 1757 [20,21,22,23,24,25,26,27]. Additional historical information based on the selection and identification of sources and the existing literature is available in this article’s Supplementary Materials. Copies of votive reliefs are shown in Figure S1.

### 1.2. Thermal Water Classifications and Potential Applications

Thermal treatment is an ancient branch of so-called “natural medicine” that is commonly employed, via different methods, for therapeutic purposes [28]; it is a treatment approach that is used in addition to conventional treatment in several countries with abundant thermal and mineral springs.

The term “thermal water” refers to natural mineral waters whose features are suitable for therapeutic use (Directive 2009/54/EC of the European Parliament and of the *Council* of 18 June 2009 on the exploitation and marketing of natural mineral waters). The therapeutic value of mineral waters has largely been studied by European balneologists. Mineral springs with different mineral contents are often recommended for specific therapeutic uses. They are classified according to their main physical and chemical characteristics (temperature, pressure, ionic concentration, radioactivity, and presence of specific ions or active chemical groups). The classification performed by Marotta and Sica dates back to 1933 and, in Italy, represents the current water classification regulations through which licenses to use mineral waters are disseminated [29]. This classification includes three parameters: temperature, fixed residue, and chemical composition [30,31,32].

While hydrotherapy (HT) generally employs ordinary tap water, in the context of medical treatment, balneotherapy (BT) utilizes thermal mineral water from natural springs, natural gases (CO_2_, iodine, sulfur, radon), peloids (mud), and other edaphic remedies (e.g., hay). It is often used for relieving chronic pain, which is a common symptom of several illnesses [33]. Additionally, BT is usually practiced in spas, where their peculiar atmosphere is part of a complex therapeutic program, which is why the term is often used synonymously with spa therapy [34].

Thermal water is generally used at a temperature between 34 °C and 36 °C. Water reduces the severity of pain in rheumatic joints, as the hydrostatic force relieves pain and reduces loading. The buoyancy and warmth of water may block nociception by acting on thermal and mechano-receptors. Warm water can also enhance blood flow, thus helping in dissipating algogenic chemicals and facilitating muscle relaxation [35]. Indeed, it has been described that immersion in thermal mineral water or the application of mudpacks may have beneficial effects on muscle tone, joint mobility, and pain intensity [36]. Increases in buoyancy and hydrostatic pressure during immersion in thermal water cause many physiological changes. Immersion up to the suprasternal notch in spa water (35 °C) results in a cascade of reactions, including increased diuresis, natriuresis, and cardiac output. Since sulfur spa baths have been successfully used in various skin immune-mediated afflictions, it has been suggested that the absorption of the trace elements present in mineral water and mudpacks through the skin may affect the immune system [35].

## 2. Nitrodi’s Spring Water: Advances and Opportunities

In addition to conventional treatment, natural products (thermal water, plants, and minerals) have played a key role in drug discovery, not only in dermatological and rheumatic diseases but also in other therapeutic areas [37,38,39,40]. Both balneological and hydroponic therapies at ‘the oldest spa in the world’, namely Nitrodi’s spring, have proved effective with respect to several disorders and conditions.

The Nitrodi’s station is located in the village of Buonopane in the south of Ischia Island and is surrounded by verdant hills. The park winds along characteristic terraces that follow the form of the land in accordance with local rural traditions. The Nitrodi’s spa complex has showers and washbasins, which issue pure water directly from the springs without chemical storage or physical treatment of any sort and at its natural temperature of 28 °C. There are comfortable dressing rooms, waterfall showers, washbasins, chairs, and beach umbrellas in sections of the park. Health management is ensured according to the current legislation (Legge Regionale 29 luglio 2008, n. 8. “Disciplina della ricerca ed utilizzazione delle acque minerali e termali, delle risorse geotermiche e delle acque di sorgente, Art. 24 Direzione sanitaria nelle aziende termali, negli stabilimenti termali e nei reparti termali). Nitrodi’s thermal baths host from 25,000 to 30,000 customers per year. Clients can spend short periods of 1–2 days there or, in several cases, they come and stay for longer treatments and therefore spend periods of 10–12–15 days there. Psoriasis, dermatitis, wounds, and ulcers are the most frequent pathologies treated. There are no standard or personalized therapeutic programs; therapy consists of washing via taking showers without any contraindication. Nitrodi also has an aromatherapy itinerary, which gives visitors the chance to come into direct contact with its aromatic plants and medicinal herbs, thus reinforcing the effect of its waters.

We aimed to investigate how Nitrodi’s water affects the inflammatory microenvironment and exerts its biological properties. Therefore, in this study, we present the results from our own studies and that of other studies in order to highlight the advances pertaining to Nitrodi’s water and the opportunities arising from its clinical application.

### 2.1. The Physical, Chemical, and Biological Characteristics of Nitrodi’s Water

Over time, the water from Nitrodi’s spring has been subjected to various official analyses. The first analysis was conducted in 1984, and the findings were confirmed by the University of Naples Federico II in an analysis recognized by the Italian Ministry of Health (Decree no. 3509, 9 October 2003). Our group recently undertook an experimental project on Nitrodi’s water wherein additional chemical and chemical–physical analyses were performed [41], the main results of which are reported in Table 1.

In addition to analyses of the water’s chemical composition and physical characteristics, evaluations of its Polycyclic Aromatic Hydrocarbons (PAHs), Organochlorines (OCLs), Organophosphorus Pesticides (OPPs), Polychlorinated Biphenyls (PCBs), and Trihalomethanes (THMs) were conducted through extraction and mass spectrometry analyses. All analyte concentration values were below the Limit of Quantification (LOQ). Future projects will include biological characterization and an analysis of the microbiome of Nitrodi’s water. In fact, Nitrodi’s water was sterilized to perform in vitro experiments with epithelial cells, thus alleviating the risk of microbial contamination. This strategy allowed our group to analyze only the effects of soluble factors (not yet known) and mineral components (reported in Table 1) on cell activities. Therefore, the results obtained to date reproduce only a part of the beneficial effects of Nitrodi’s water.

Nitrodi’s water was categorized as mildly mineralized and hypothermal (20–30 °C) based on its chemical composition and temperature, as proposed by Nasermoaddeli and Kagamimori’s classification [33]. Additionally, as proposed by Marotta and Sica in the most well-known classification in Italy [29,42], as well as by Mancioli [43], the results indicated that Nitrodi’s water falls into the category of medium-mineral water, meaning that it essentially consists of sulfate-alkaline. Regarding its clinical use, it is considered a bicarbonated water, although it does not completely meet the European Union’s requisites for such natural mineral water. It is also alkaline-earthy and naturally hypothermal [29,33,41,42,43]. Our analysis of its physical and chemical composition allows us to speculate on the beneficial properties of Nitrodi’s water. In particular, the presence of bicarbonate ions and sulphates could be important for hydropinotherapy in patients suffering from pain and other symptoms caused by biliary dyskinesias and biliary sand or following a cholecystectomy [44]. Bicarbonate mineral water improves skin regeneration by increasing keratinocyte proliferation and migration and inducing collagen and elastic fibers in the dermis [45]. Bicarbonate ions are also useful for musculoskeletal rehabilitation as they reduce the effects of muscular exertion. Its sodium and silica content could be responsible for the beneficial effects of Nitrodi’s water on skin disorders, as already demonstrated by different research groups [46].

There is a growing interest in the application of Nitrodi’s water as an active ingredient in cosmetics (unpublished data). The richness of the bicarbonate and the mixture of minerals contained in Nitrodi’s water favor natural skin exfoliation, i.e., peeling, thus removing damaged and aged skin [47]. Exfoliation induces keratinocyte and fibroblast migration, structural reorganization, and the deposition of new collagen, which are all effects induced by Nitrodi’s water [41].

It is also conceivable that the therapeutic effects of Nitrodi’s water could be associated with the native microbiome. Interestingly, Nicoletti et al. have demonstrated that Comano spring water-derived bacterial lysates exert regenerative effects on human skin fibroblasts. Similar to Nitrodi’s water, Comano’s water, found in the province of Trento (Northern Italy), is bicarbonate-calcium-magnesium-based and recommended for cutaneous disorders and upper airway diseases [48]. Nitrodi’s water is certified as microbiologically pure as it contains no pathogenetic microorganisms. However, the anti-inflammatory and regenerative properties of these waters cannot be solely traced back to their mineral composition but the combination of their mineral composition and specific bacterial species. Therefore, studying Nitrodi’s microbiome is necessary to better understand the therapeutic bases of these waters.

### 2.2. Beneficial Effects of Nitrodi’s Water: Data from the Past

The effects of Nitrodi’s spring water were extensively studied in the late 1960s to evaluate the different modalities of exposure to the water. These studies showed that both internal and external use yielded several benefits in the treatment of certain disorders and in physiological processes such as wound healing [43].

In 1984, Mancioli published ‘posthumous’, a research publication commissioned by the Center Studies on the Island of Ischia, which was carried out over a three-year period between 1968 and 1970 at the Study Center «P. Malcovati Lacco Ameno Terme» on behalf of the Order of Merit of Labor Angelo Rizzoli. The studies were intended to ascertain, in a modern scientific sense, the therapeutic validity of using Nitrodi’s spring water in view of the realization of a grandiose valorization project.

In the first part of the present manuscript, an extensive analysis of the properties of Nitrodi’s water, based on the studies performed in 1969 by Prof. M. Talenti from the Institute of Hygiene of the University of Rome, was performed.

Water is one of the most important stimulators of diuresis, and in some waters, this property acquires a clearly higher value than that of ordinary waters. With this in mind, Mancioli addressed the initial experiments that evaluated the ability of Nitrodi’s spring water to modify the diuresis of normal subjects, and this was compared to water from the aqueduct, which was considered the control.

Although it was not easy to create a defined limit between common drinking water and diuretic waters, through reviewing the evidence, the author recognized the ability of Nitrodi’s spring water to (i) cause polyuria and (ii) induce polyuria in a more intense and quicker way compared to common drinking water. The results of Mancioli’s analysis showed that the diuresis caused by Nitrodi’s water was more intense and, above all, took place much more rapidly than what occurred after the ingestion of the aqueduct water. The author also demonstrated that the diuretic action of Nitrodi’s mineral water gradually increased with the repeated consumption of the water for several consecutive days compared to the control. The acid uric turnover was likewise evaluated, showing that Nitrodi’s water was able to markedly decrease uricoemia. Changes in the blood and urine levels of uric acid observed during the treatment of Nitrodi’s water suggested the ability of this water to mobilize uric acid from tissue deposits [43].

Mancioli also evaluated the effects of the water in the context of gastritis and subacute and chronic gastroduodenitis. Patients suffering from gastric and duodenal ulcers visited Nitrodi’s spring spontaneously and reported an undeniable benefit from drinking the water. Furthermore, Nitrodi’s water was found to be singularly effective in the topical treatment of varicose ulcers, numb sores, sinus tracts, etc., confirming an ancient local empirical tradition [43].

**Figure 1:**
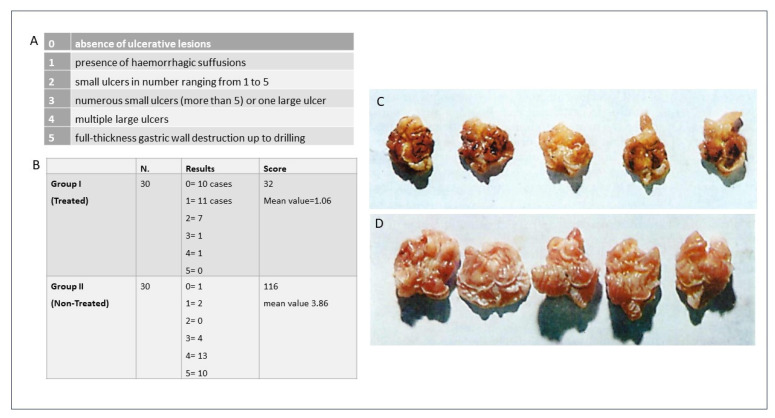
Classification of the extent of the lesions (**panel A**); experimental model (**panel B**); qualitative analysis of the macroscopic findings of the stomachs of rats treated with aqueduct water (**panel C**) and Nitrodi’s water (**panel D**). Courtesy of Li Causi Editore; Bologna, 1984.

To this end, in the final series of experiments conducted by the author, 60 albino female Spreague Dawley rats were treated in an in vivo model to evaluate the efficacy of the Nitrodi’s spring water. The animals were divided into two groups: those treated for 15 consecutive days with Nitrodi’s water twice a day (30 cc/kg of weight) and those treated using equal amounts of water from the aqueduct. At the end of the treatment stage, an experimental ulcer was induced via the intraperitoneal injection of reserpine at a dose of 0.5 mgr/kg. Approximately 16 h after the intraperitoneal injection of reserpine, the animals were sacrificed, and their stomachs were removed via laparotomy. To evaluate the extent of the lesions found and to establish a criterion of comparison between the various findings, the author followed a classification according to an arbitrary scale. The histological exam demonstrated that, in the animals previously treated with Nitrodi’s water, compared to the control group, the ulcers affected only the most superficial layers of the mucosa and rarely involved the muscularis mucosae. Furthermore, a qualitative analysis showed the scarcity of the lesions, as they were mostly expressed as hemorrhagic suffusions or superficial ulcerations in the animals treated with Nitrodi’s water (Figure 1).

## 3. Beneficial Effects of Nitrodi’s Water: Ongoing Data

Recently, among scientists and clinicians, interest in the properties of Nitrodi’s water has grown. Although many clinical and experimental data are still unpublished, we believe that the knowledge of this water will soon develop and that more potential applications will arise. In 2020, we decided to confirm some of the evidence described by Mancioli, expand the knowledge of the effects of this water, and elucidate some of the biological mechanisms sustaining the therapeutic efficacy of Nitrodi’s water, providing a robust scientific basis that could confirm its usage in hydroponic and balneological therapy.

### 3.1. Anti-Inflammatory and Anti-Oxidant Effects of Nitrodi’s Water

Inflammation represents the immune system’s response to infection with foreign organisms and injury [49] and underlies a wide variety of physiological and pathological processes such as arthritis, cancer, atherosclerosis, and neurodegenerative diseases [49].

After wounding, the first phase begins with vascular constriction and fibrin clot formation. Once bleeding is controlled, inflammatory cells migrate to the wound (chemotaxis) and initiate the inflammatory phase. The first responders are neutrophils, activated by pro-inflammatory mediators released by damaged tissue and by Damage-Associated Molecular Pattern molecules (DAMPs). Neutrophils remove foreign material from the wounds. During this process, substances such as reactive oxygen species (ROS) and proteases are generated by neutrophils, causing some additional damage [50]. Next, immune cells arrive at the scene, including monocytes that differentiate into macrophages and coordinate the wound healing process. As the inflammation subsides and the number of leukocytes diminishes, the wound undergoes a lengthy period of remodeling and resolution, which is mediated by fibroblasts and keratinocytes. Certain medical conditions, mainly diabetes or immunosuppression, can have negative impacts on the wound healing response. There remains an urgent need to develop effective alternative therapeutics that can be used in combination with conventional treatments in order to improve wound healing and resolve impaired wounds.

**Figure 2:**
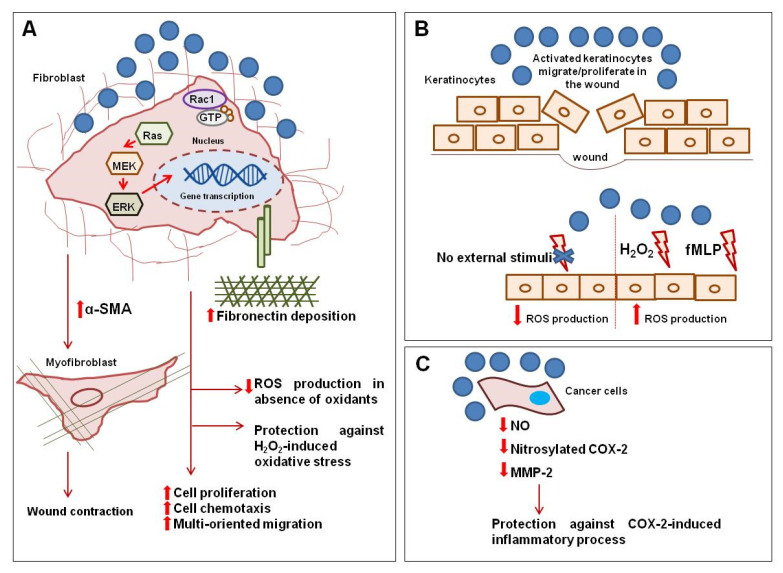
Overview of the in vitro biological effects of Nitrodi’s water in tissue repair and stress-related processes. (**A**) The treatment of dermal fibroblasts with filtered Nitrodi’s water induces the expression of the extracellular signal-regulated protein kinases (ERK) involved in cell growth and cell-cycle progression. Nitrodi’s water favors the transition of fibroblasts to myofibroblasts, inducing alpha-SMA expression. In tissue repair, myofibroblasts are crucial for the deposition of ECM proteins and wound contraction. Fibroblasts treated with Nitrodi’s water exhibit lower levels of reactive oxygen species (ROS) both in basal conditions and in the presence of oxidants. (**B**) The treatment of keratinocytes with Nitrodi’s water induces cell proliferation and migration, which are crucial for wound healing. Nitrodi’s water reduces ROS levels in the absence of oxidant stimuli but not in response to hydrogen peroxide (H_2_O_2_) and bacterial-derived peptides (fMLPs). (**C**) The downregulation of protein S-nitrosylation can be observed in cancer cells treated with Nitrodi’s water. In particular, the downregulation of COX-2 and its target MMP-2 is induced by Nitrodi’s water in colorectal adenocarcinoma cells. Blue circles indicate cell treatment with Nitrodi’s water; red arrows indicate the effects of Nitrodi’s water on cellular processes; double red arrows indicate an increase or decrease in protein expression or cellular activities.

Based on our long-standing experience in the study of allergic and autoimmune inflammatory processes [51,52,53,54,55,56,57,58,59,60], an experimental strategy using different in vitro techniques was set up to assess the effects of Nitrodi’s spring water in many aspects associated with low-grade inflammation and stress-related conditions. The results from these experiments are illustrated in Figure 2.

### 3.2. Cell Treatment with Filtered Nitrodi’s Water

Firstly, the phenomenon of S-nitrosylation, understood as the addition of nitric oxide (NO) to the thiol side chain of cysteine residues within proteins, has been the subject of a lot of academic interest. The aberrant S-nitrosylation of proteins is associated with many diseases [61]. NO is also a key messenger in the pathogenesis of inflammation and exerts this effect by activating innate and adaptive immunity. Cyclooxygenase-2 (COX-2) expression has pathological significance; COX-2 could be activated by S-nitrosylation inducing MMP-2, which is responsible for the degradation of the ECM. To analyze the effects of Nitrodi’s water on protein S-nitrosylation, the expression of MMP-2 was investigated in colorectal adenocarcinoma cells (RKO cell line). The results obtained from these experiments showed that MMP-2 protein expression and protein S-nitrosylation were markedly downregulated in RKO cells treated with Nitrodi’s water. Overall, the results revealed that the molecular mechanisms through which Nitrodi’s spring water exerts its anti-inflammatory effects might involve the downregulation of protein S-nitrosylation [61].

Recent evidence has shown that oxidative stress plays a pivotal role in the development and perpetuation of inflammation [62]. Oxidative stress implies increased intracellular ROS levels, which are involved in both chronological aging and photo-aging [41]. Promising results have been obtained from using natural antioxidant compounds as pharmaceutical tools against several inflammatory diseases in in vitro systems, in vivo models, and in clinical trials. Napolitano et al. showed that Nitrodi’s water is a promising source of natural antioxidants that could potentially be useful in chronic inflammation and anti-aging strategies. Treatment with these waters reduced the basal production of ROS in a model of dermal fibroblasts, namely BJ cells. These data were also confirmed in other models of epithelial cells, or rather keratinocytes. In response to stress-associated stimuli, such as H_2_O_2_, the levels of ROS in the treated cells were lower compared to the untreated cells, suggesting that Nitrodi’s water is able to contain the damage caused by oxidative stress in dermal fibroblasts. Instead, Nitrodi’s water was not able to reduce ROS release in response to H_2_O_2_ in keratinocytes. This behavior can be explained by the fact that ROS are used by keratinocytes to communicate with the underlying cells in order to promote wound repair. Since ROS also operate as intracellular signaling molecules in response to external stimuli, it has been demonstrated that Nitrodi’s water does not affect ROS generation in response to bacterial-derived peptides [41].

### 3.3. Regenerative Properties of Nitrodi’s Water

Topical treatment with thermal waters exerts regenerative action on skin probably due to the favorable combination of a local wet environment and an anti-inflammatory effect. In vitro studies have demonstrated that Nitrodi’s water exerts a positive effect on dermal fibroblast and keratinocyte activities.

In particular, the expression of extracellular signal-regulated protein kinases 1 and 2 (ERK 1/2), which are involved in growth and cell-cycle progression, was induced in dermal fibroblasts via treatment with Nitrodi’s water. Nitrodi’s water can sustain dermal fibroblast survival both in starving and growing conditions [41].

Dermal fibroblast migration is a pivotal step in the healing process. Nitrodi’s water induced both chemotaxis and wound-scratch closure in dermal fibroblasts.

Upon injury, dermal fibroblasts migrate into wound granulation tissue and differentiate into myofibroblasts, which play a pivotal role in the wound contraction and deposition of ECM proteins. Alpha smooth muscle actin (alpha-SMA) expression is used as a marker of myofibroblast differentiation [63]. Nitrodi’s water can induce alpha-SMA expression in dermal fibroblasts, thus promoting fibroblast–myofibroblast transition.

The ECM is crucial for structural support and cellular attachment, regulating biochemical pathways and directly modulating cell adhesion, proliferation, and migration. The healing process occurs through the formation of the synthesis of the ECM, much of which is due to fibroblasts. In the newly formed ECM, all of the ECM proteins are created, with the exception of elastin [62]. Treating dermal fibroblasts with Nitrodi’s water promotes the deposition of collagen type I and fibronectin, which are major ECM proteins that are essential for both cell adhesion and structural support [64].

Keratinocytes play a key role in ensuring that the wound healing process is properly carried out through coordinated action with fibroblasts and immune cells. Cells can migrate, proliferate, and differentiate, contributing to the re-epithelialization of epidermal tissue [65]. These processes are impaired in all types of chronic wounds. Treatment with Nitrodi’s water induces keratinocyte proliferation and chemotaxis.

Co-culture experiments are being set up in order to study keratinocyte–fibroblast interactions in the wound healing process (unpublished data). In fact, there is a large body of evidence to suggest that keratinocytes stimulate fibroblasts to synthesize growth factors, which in turn stimulate keratinocyte proliferation in a double-paracrine manner. Importantly, fibroblasts can acquire a myofibroblast phenotype under the control of keratinocytes [65]. The results from these experiments will have a major impact on both clinical practices and the cosmetics industry.

Overall, these in vitro data are scientific proof of the beneficial effects Nitrodi’s water has on the skin. The anti-inflammatory, anti-oxidant, and regenerative effects of Nitrodi’s water that have been observed in in vitro studies are shown in Figure 2.

### 3.4. Future Perspectives and Potential Applications in Clinical Practice

Long regarded as an alternative treatment in European countries, balneotherapy (BT) was one of the first non-pharmacological treatments evaluated in different acute and chronic diseases.

The mechanisms by which BT acts in the treatment of autoimmune diseases are not fully understood. While this therapy does not replace but rather supplements traditional drug therapy, it is certainly beneficial in appropriate cases [66,67].

BT is defined as the use of mineral water, gases, or peloids for health promotion and the prevention or treatment of diseases. In a Hungarian double-blind, controlled pilot study, Tiszasüly and Kolop mud packs were shown to have a favorable effect in the treatment of knee osteoarthritis. Indeed, Tiszasüly and Kolop peloids are inorganic because of their notable mineral content and considerable lack of organic components [68]. A systematic review on the beneficial effects of different BT modalities, including radon–carbon dioxide baths, mud packs, hot sulfur baths, and Dead or Red Sea baths for patients with RA, has been published [69]. In 2018, Fioravanti et al. demonstrated the efficacy of BT in patients with fibromyalgia by using the thermal water of Vetriolo (Trento, Italy), which descend from the Vetriolo spring in the thick of the fir woods at an altitude of 1500 m. Indeed, Vetriolo’s water is a highly mineralized (fixed residue at 180 °C 1702 mg/L) strongly acidic (pH 5.7) sulfate (1100 mg/L) water that is rich in calcium (111.0 mg/L), magnesium (65.5 mg/L), and iron (315 mg/L) [70]. Several studies have indicated the efficacy of BT with natural mineral water for psoriasis and atopic dermatitis [71].

BT plays a role in the complex, multimodal therapy of rheumatic diseases; therefore, the American College of Rheumatology has positioned it as an essential complementary therapy for rheumatic diseases [72].

Important features of BT include the temperature and composition of the mineral water used, which depends on the respective country. Mudpacks or peloids can be applied wet or dry (in the form of sand), and their properties depend on their origin. Waters can be enriched with gases, mainly CO_2_ or radon. All these types of BT usually take the form of baths for the whole body or the affected portion of the body [73]. The benefits of BT include improved motion and reduced stiffness and pain. These benefits may have a positive impact on RA symptoms, which could improve the quality of life of these patients [73,74,75].

Although the experimental evidence described in the present review collectively suggests that Nitrodi’s water exerts in vitro regenerative action by promoting wound healing, in vitro wound healing assays cannot mimic the complexity of the conditions that occur during the in vivo wound healing process. Hence, data obtained from in vitro assays should be corroborated with in vivo models. Therefore, we are planning a large observational prospective study to evaluate the effects of crenotherapy on wound repair and regeneration using Nitrodi’s thermal water in a model digital ulcer (DUs) from patients affected by SSc over a 12-month follow-up period. We will perform tests such as the Scleroderma Health Assessment Questionnaire (SHAQ), pain and fatigue VAS, SF36 (Quality of Life–QoL), HAQ (General Disability). In addition, we will assess the overall DU number and reduction in new DUs, as well as some qualitative parameters, such as the presence of infections, presence of devitalized tissue (necrosis/fibrin), amount of exudates, presence of granulation tissue, and epithelization of the wound. Finally, we will evaluate morphology microcirculation using nailfold capillaroscopy [60,76]. These findings could contribute to developing novel strategies that aim to complement conventional treatments by using Nitrodi’s water as a potential treatment for skin diseases and age-related skin deterioration.

## 4. Conclusions

Studies on the cellular biology of the properties of thermal waters in the world are limited. Italy represents a unique case in that it is particularly rich in springs that share similar therapeutic effects and have similar salt and thermal features to that of Nitrodi’s spring water, probably because they have the same hydro-geological origin.

The present review presents the results of all of the in vitro studies performed to confirm the therapeutic role of Nitrodi’s water. The elucidation of the biological mechanisms underlying the benefits of Nitrodi’s water will contribute to the development of potential therapies for skin diseases. Nitrodi’s water could be a promising anti-inflammatory agent for the skin and a potential wound-healing therapeutic agent. In addition, the antioxidant properties of Nitrodi’s water could be exploited to prevent symptoms related to photo-induced skin aging.

These results concur with all the previously reported therapeutic properties of Nitrodi’s spring water and thus reinforce the concept that the natural resource is an important complementary therapy to traditional medicine.

## Figures and Tables

**Table 1:** Chemical and chemical–physical analysis of Nitrodi’s spring water.

Parameters	Unit	Results
Water temperature at source	°C	+28.4
pH at source	/	6.33
Silica (SiO_2_)	mg/L	82
Bicarbonate (HCO_3_^−^)	mg/L	561
Chlorides (Cl^−^)	mg/L	93
Sulphates (SO_4_^2−^)	mg/L	204
Sodium (Na^+^)	mg/L	174
Potassium (K^+^)	mg/L	21
Calcium (Ca^2+^)	mg/L	137
Magnesium (Mg^2+^)	mg/L	17
Iron (dissolved) (Fe^2+^, Fe^3+^)	mg/L	<0.02
Ammonium (NH_4_^+^)	mg/L	<0.02
Phosphorus (P total)	mg/L	<0.05
Stronzium (Sr^2+^)	mg/L	0.31
Lithium (Li^+^)	mg/L	0.04
Aluminum (Al^3+^)	mg/L	<0.02
Bromide (Br^−^)	mg/L	0.18

## Data Availability

No new data were generated or analyzed for this review article.

## Supplementary Materials

The following supporting information can be downloaded at: https://www.mdpi.com/article/10.3390/ijms241813685/s1.
