# Supplementary material for: The Benefits of Water from Nitrodi’s Spring: The In Vitro Studies Leading the Potential Clinical Applications
Source: Int J Mol Sci. 2023 Sep 5;24(18):13685. doi: 10.3390/ijms241813685 (PMC10531131; doi:10.3390/ijms241813685)
Supplement: Supplementary file 1 [file ijms-24-13685-s001.zip › ijms-2553747-supplementary.pdf]

International Journal of *Molecular Sciences*

MDPI

Review

The Benefits of Water from Nitrodi's Spring: The In Vitro Studies Leading the Potential Clinical Applications

Ilaria Mormile [1,†], Fabiana Tuccillo [2,†], Francesca Della Casa [1], Valentina D'Aiuto [3], Nunzia Montuori [1,4], Marina De Rosa [5], Filomena Napolitano [1,*], Amato de Paulis [1,4] and Francesca Wanda Rossi [1,4]

1 Department of Translational Medical Sciences, University of Naples Federico II, 80131 Naples, Italy; ilariamormile87@gmail.com (I.M.); francescadellacasa4@gmail.com (F.D.C.); nmontuor@unina.it (N.M.); depaulis@unina.it (A.d.P.); francescawrossi@gmail.com (F.W.R.)
2 Department of Law, University of Naples Federico II, 80138 Naples, Italy; fabiana.tuccillo@unina.it
3 Post-Graduate Program in Clinical Immunology and Allergy, University of Naples Federico II, 80131 Naples, Italy; valedaiuto@tiscali.it
4 Center for Basic and Clinical Immunology Research (CISI), WAO Center of Excellence, University of Naples Federico II, 80131 Naples, Italy
5 Department of Molecular Medicine and Medical Biotechnology, University of Naples Federico II, 80131 Naples, Italy; marina.derosa@unina.it
* Correspondence: filomena.napolitano@unina.it; Tel.: +39-(081)-7463611
† These authors contributed equally to this work.

Citation: Mormile, I.; Tuccillo, F.; Della Casa, F.; D'Aiuto, V.; Montuori, N.; De Rosa, M.; Napolitano, F.; de Paulis, A.; Rossi, F.W. The Benefits of Water from Nitrodi's Spring: The In Vitro Studies Leading the Potential Clinical Applications. *Int. J. Mol. Sci.* **2023**, *24*, 13685. https://doi.org/10.3390/ijms241813685

Academic Editor: Philip W. Wertz

Received: 27 July 2023
Revised: 28 August 2023
Accepted: 29 August 2023
Published: 5 September 2023

Copyright: © 2023 by the authors. Licensee MDPI, Basel, Switzerland. This article is an open access article distributed under the terms and conditions of the Creative Commons Attribution (CC BY) license (https://creativecommons.org/licenses/by/4.0/).

Supplementary Material

Baths in the Roman culture

In the Mediterranean area exploitation of thermo-mineral water springs is an antique phenomenon [1]. Since ancient times water becomes charged with a series of positive values in its relationship to man [2]. Herodotus speaks of a divine water healing to all diseases, including blindness, deafness, and dumbness [3]. In Italy, an ample number of springs supported bathing establishments and infrastructures that date back to the pre-Roman age, left on the territory deep, and sometimes still well-visible marks that often represent the basis of modern thermo-mineral resorts.

Campania is one of the richest regions in thermal and mineral water resources [4]. It may be said that Romans revalued thermalism. In the Roman age, bathing was a social and recreational activity deeply rooted in daily life. There are numerous waters with special chemical and physical properties and declared healthful features, which allowed, since Roman times (VIII - V centuries BC), their use for therapeutic purposes and human consumption. Thermalism was developed by the Romans, by building first waterworks and thermal structures in the cities, and then large spas in Baia, Campi Flegrei, Agnano, Ischia [5], and many other locations, that were highly praised for the cure of a wide range of pathologies, due to the volcanic origin of their waters and their diversified physical-chemical and thermic properties. Thanks to its peculiar features, thermalism continued to be highly valued also after the end of the Empire [6,7], when the urban crisis, the destruction of part of the aqueducts and the decay of the thermal structures [8] caused the irreversible end of hydrotherapy in the artificial urban spas but not in the natural ones (such as those in Ischia), that continued to attract many pilgrims patients during the Middle Ages and the Renaissance.

Going to baths was a central event in the Roman culture and a very important part of the daily life [9–11], especially when Romans realized the health benefits brought out by water [12–14]. Romans developed and perfected heating and water supply technologies, thus changing bathing from a simple hygienic practice to a pleasurable activity and a recommended medical therapy. The program of bathing involved a stepwise progression from cold to hot areas and included sweating, massage, and a final immersion into a cold pool. The average Roman spent a considerable part of the day in public baths: bathing in spa was indeed an activity that incorporated sports, games, massage, body care and

relaxation [15–18], and medical treatments, much like a social club or a community center. In many spas there were facilities such as libraries and lecture halls as well as galleries and exedrae for the exhibition of works of art.

Thermalism assumed a twofold function: therapeutical, with medical and control centers, and social [19,20], with separate areas for men and women, where they could relax, study, and meet other people. It was thus realized what Juvenal wrote in his Satires (10.56: orandum est ut sit mens sana in corpore sano): a healthy mind in a healthy body.

In Nitrodi's spring, already known to the first Greek settlers in southern Italy and dedicated to Apollo and the Nitrodi Nymphs, everyone found a remedy: war wounds healed in a short time, old men became looser, and women emerged from the water more beautiful. Thus, the thermal waters of Nitrodi became a very important cult center between the 1st century BC and the 3rd century AD. The healing power of Nitrodi's water was considered a gift from the nymphs and the god Apollo. As evidence of that ancient cult, today we can admire the votive reliefs in the Archaeological Museum of Naples, found in Nitrodi in 1757. Towards the end of the 19th century, Mommsen in the tenth volume of the *Corpus Inscriptionum Latinarum* [21], in the section dedicated to epigraphs from Campania under the heading *Aenaria insula* referred: *«in insula Ischia in loco adhuc dicto Nitroli in valle Foriae prope Serraram, ubi thermae sunt, sacrarium Apollinis et Nympharum Nitrodarum eruderatum esse et tredecim inde anaglypha illata in museum publicum Neapolitanum».*

The first discovery of ex-votos was made by Doctor Giovanni Pistoja, who was in Ischia for spa treatments in the second half of the 17th century and was present at their occasional discovery by some boys [22]. A relief and a small altar were unearthed. The doctor intended to take them to Naples, but they were later lost during transport to the mainland. The drawings of these two finds are known from memory and are present in some copies of the Giulio Jasolino's volume entitled *De' rimedj naturali che sono nell'Isola di Pithecusa, hoggi detta Ischia* - Napoli 1689. The physician Jasolino described the nature of the Nitroli waters (this name remained in use until the first half of the 20th century, then replaced by the more recent Nitrodi) [23,24], used by the inhabitants of Barano with the preservation of their health, considering it a balm to nourish and preserve their bowels, referring to the digestive-diuretic properties, its warm spring, and its notable benefit to make urination. He believes is due to some Nitro mine found there, from which it may have taken the name Nitroli [22].

The second group of votive reliefs was discovered in 1757 and consists of 13 elements [25]. The reliefs, according to the numbering of the room named Pithecusa in the Archaeological Museum of Naples with the inscriptions, are analysed by S. Iapino, [26,27]. The reliefs depict the god Apollo, who was the god who protected health, and the Nymphs who embodied the 'means' through which to obtain and maintain the benefits achieved from the spring source of Nitrodi. Of these only 11 have come down to us. One of these was lost as early as 1845-46. The other, dedicated to Menippus, Apollo, and the Nitrodi Nymphs, was kept in the National Archaeological Museum in Naples until 1989 and was later stolen during work on some rooms in the museum (Figure S1).

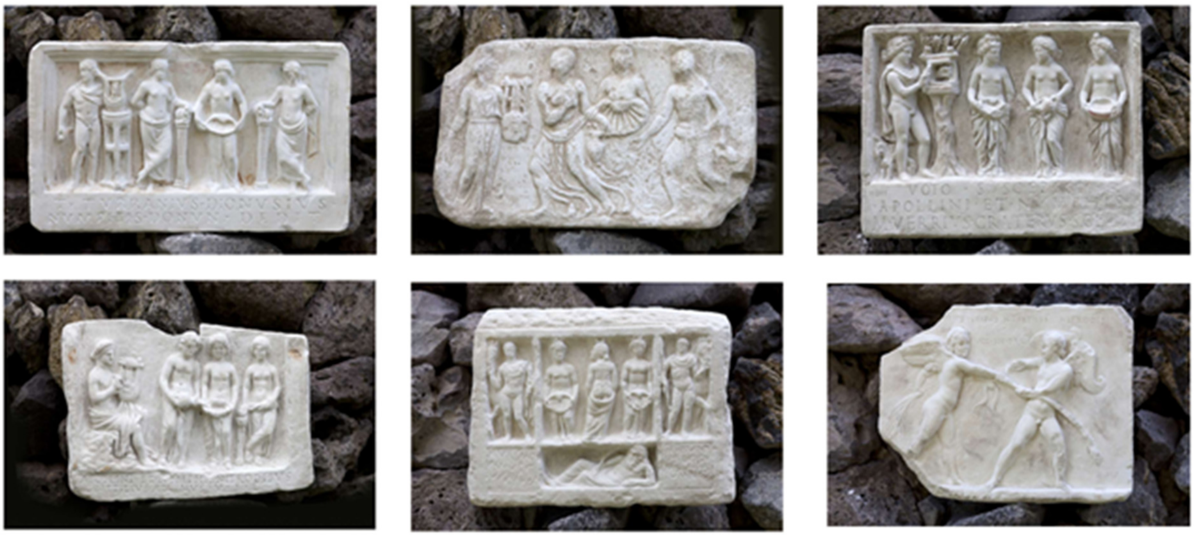

Figure S1. Copies of votive reliefs. With courtesy of Giuseppe Di Meglio (Fonti delle Ninfe Nitrodi, via Pendio Nitrodi, 80070 Barano d'Ischia) who supplied pictures.

There is also another relief, in the collection of Lyde Browne in Wimbledon, which, following the purchase of the entire collection by Catherine II in 1785-87, is now in the Hermitage Museum in St. Petersburg [21,28]. The Russian scholar O. Neverov attributes it to the collection of votive tablets found at the Nitrodi spring in 1757. It is a marble slab with the image of three nymphs and epigraphic text distributed between upper and lower frame [27].

The possible interpretation or 'decipherment' that one wishes to give of these sculpted marble slabs is made more important considering the continuity of attendance from Greek and Roman times to the present day of the natural spring, to whose waters therapeutic-medicinal properties have always been attributed.

References

1. Zanetti, C. Acque e siti termali nell'Italia romana le testimonianze degli autori antichi. In Proceedings of the Acque e siti termali nell'Italia romana le testimonianze degli autori antichi, in Aquae Patavinae, Montegrotto, Italy, 6–8 September 2012; Aggiornamenti e nuove prospettive di valorizzazione: Padova University Press: Padova, Italy, 2012; p. 365.
2. Sorcinelli, P. *Storia Sociale dell'acqua*; Riti e culture: Milan, Italy, 1998.
3. Sunseri, G.B. Herod. 2.13.3. Sul controllo e l'uso delle acque in Erodoto. Spunti di riflessione. *ὀρμος. Ric. Di Stor. Antica* **2018**, *10*, 203. Available online: https://www.perseus.tufts.edu/hopper/text?doc=Perseus%3Atext%3A1999.01.0125%3Abook%3D2%3Achapter%3D13%3Asection%3D3 (accessed on 4 September 2023).
4. Fagan, G.G. The Genesis of the Roman Public Bath: Recent Approaches and Future Directions. *Am. J. Archeol.* **2001**, *105*, 421.
5. Plin. Naturalis historia. *31.5.9*. Available online: https://la.wikisource.org/w/index.php?title=Naturalis_Historia/Liber_XXXI&action=edit&section=5 (accessed on 4 September 2023).
6. Brundrett, N.G.R., Simpson, C.J. Innovation and the Baths of Agrippa. *Athenaeum* **1997**, *87*, 220.
7. Di Capua, F. *L'idroterapia ai Tempi dell'impero Romano*; Istituto di studi Romani: Rome, Italy, 1940.
8. Malissard, A. *Les Romains et l'eau. Fontaines, Salles de Bains, Thermes, Egouts, Aqueductus*; Les Belles Lettres: Paris, France, 1994.
9. Brödner, E. *Die Römischen Thermen und das antike Badewesen: Eine kulturhistorische Betrachtung Darmstadt*; Wissenschaftliche Buchgesellschaft Darmstadt, Germany, 1983.
10. Pasquinucci, M. *Terme Romane e Vita Quotidiana*; Franco Cosimo Panini: Modena, Italy, 1987.
11. Nielsen, I. *Thermae et Balnea. The Architecture and Cultural History of Roman Public Baths*; Aarhus University Press: Aarhus, Denmark, 1990.
12. Plin. Naturalis historia. 31.2.5. Available online: https://la.wikisource.org/w/index.php?title=Naturalis_Historia/Liber_XXXI&action=edit&section=2 (accessed on 4 September 2023)
13. Vitr. *8.3.5*. Available online: https://www.thelatinlibrary.com/vitruvius/vitruvius8.html#3.5 (accessed on 4 September 2023)

14. Sen. Naturales Quaestiones. *3.2.1*. Available online: https://www.thelatinlibrary.com/sen/sen.qn3.shtml (accessed on 4 September 2023)
15. Jackson, R. Waters and Spas in the Classical World. *The Medicinal History of Waters and Spas. Porter Edition (London).* **1990**, *10*, 1–13.
16. Staccioli, A. *Le terme di Roma antica*; Newton Compton Editori, Rome, Italy, 1995.
17. Fagan, G.G. *Bathing in Public in the Roman World Ann Arbor*; University of Michigan Press: Ann Arbor, MI, USA, 2002.
18. Pettenò, E. Acque termali e uso terapeutico del bagno nel mondo romano. In Proceedings of the Termalismo antiguo I Congreso Peninsular, Actas, Arnedillo, Spain, 3–5 October 1996, UNED: Madrid, Spain, 1997.
19. Perex Agorreta, M.J. *Termalismo antiguo I Congreso Peninsular*; Casa de Velazquez: Madrid, Italy, 1997; p. 217.
20. Muscettola, S.A. Gli ex voto alle ninfe di ischia: La parabola di una cultura marginale. *RIA 57* **2002**, *42 nt. 24*.
21. Jasolino, G. *De' Rimedj Naturali che sono nell'Isola di Pithecusa Naples*; Bartolomeo Roselli, Napoli, Italy, 1763 Available online: https://books.google.it/books?id=R3JAAAAAcAAJ&printsec=frontcover&hl=it&source=gbs_ge_summary_r&cad=0#v=onepage&q&f=false (accessed on 4 September 2023)
22. Angeletti, R. Usi terapeutici delle acque nella trattatistica medica della tarda antichità (secoli IV-VII d.C.). In Proceedings of the L'acqua nei secoli altomedievali, Atti delle settimane di studio della Fondazione Centro Italiano di Studi sull'Altomedioevo, Spoleto, Italy, 12–17 April 2008; Fondazione CISAM, Spoleto, Italy, 2008, p. 821.
23. Mantovanelli, L. *Acque Termali e cure Mediche, in Cura, Preghiera e Benessere. Le stazioni Curative Termominerali nell'Italia Romana*; Padova University Press: Padova, Italy, 2014.
24. Forti, L. Rilievi dedicati alle Ninfe Nitrodi. *RAAN 36* **1951**, *tab. VI–XI*, 161.
25. Iapino, S. I rilievi votivi con dedica ad Apollo e alle Ninfe Nitrodi. *La Rass. D'ischia* **2003**, *3–4*, 26.
26. Chioffi, L. Ischia in età romana: Cosa dicono le iscrizioni. In Proceedings of the Il Mediterraneo e la storia II. Naviganti, popoli e culture ad Ischia e in altri luoghi della costa tirrenica, Institutum Romanum Finlandiae, Rome, Italy, 2017.
27. CIL X 6790. Available online: https://arachne.uni-koeln.de/Tei-Viewer/cgi-bin/teiviewer.php?manifest=BOOK-ZID1323145, pg. 679; (accessed on 4 September 2023).

Disclaimer/Publisher's Note: The statements, opinions and data contained in all publications are solely those of the individual author(s) and contributor(s) and not of MDPI and/or the editor(s). MDPI and/or the editor(s) disclaim responsibility for any injury to people or property resulting from any ideas, methods, instructions or products referred to in the content.
